# miRNA-1236 Inhibits HIV-1 Infection of Monocytes by Repressing Translation of Cellular Factor VprBP

**DOI:** 10.1371/journal.pone.0099535

**Published:** 2014-06-16

**Authors:** Li Ma, Chan-Juan Shen, Éric A. Cohen, Si-Dong Xiong, Jian-Hua Wang

**Affiliations:** 1 Jiangsu Key Laboratory of Infection and Immunity, Institutes of Biology & Medical Sciences, Soochow University, Suzhou, China; 2 Key Laboratory of Molecular Virology & Immunology, Institute Pasteur of Shanghai, Chinese Academy of Sciences, Shanghai, China; 3 Laboratory of Human Retrovirology, Institut de Recherches Cliniques de Montréal, Montreal, Quebec, Canada; Chinese Academy of Sciences, Wuhan Institute of Virology, China

## Abstract

Primary monocytes are refractory to HIV-1 infection and become permissive upon differentiation into monocyte-derived dendritic cells (MDDCs) or macrophages. Multiple mechanisms have been proposed to interpret HIV-1 restriction in monocytes. Human cellular miRNAs can modulate HIV-1 infection by targeting either conserved regions of the HIV-1 genome or host gene transcripts. We have recently reported that the translation of host protein pur-alpha is repressed by abundant cellular miRNAs to inhibit HIV-1 infection in monocytes. Here, we report that the transcript of another cellular factor, VprBP [Vpr (HIV-1)-binding protein], was repressed by cellular miRNA-1236, which contributes to HIV-1 restriction in monocytes. Transfection of miR-1236 inhibitors enhanced translation of VprBP in monocytes and significantly promoted viral infection; exogenous input of synthesized miR-1236 mimics into MDDCs suppressed translation of VprBP, and, accordingly, significantly impaired viral infection. Our data emphasize the role of miRNA in modulating differentiation-dependent susceptibility of the host cell to HIV-1 infection. Understanding the modulation of HIV-1 infection by cellular miRNAs may provide key small RNAs or the identification of new important protein targets regulated by miRNAs for the development of antiviral strategies.

## Introduction

miRNAs are small non-coding RNA molecules (18–22 nucleotides) found in eukaryotic cells. miRNAs are vital post-transcriptional regulators, and the binding of miRNAs to the 3′-untranslated regions on target mRNA transcripts usually results in translational repression or target degradation [1]. Aberrant expression of miRNAs has been implicated in development and progression of many infectious diseases including HIV-1 infection [2], [3], [4], [5], [6], [7], [8], [9]. Higher serum levels of miR-122 have been recently reported as potential biomarkers for AIDS-related non-Hodgkin lymphoma [10], and disrupted expression of certain miRNAs by HIV-1 or simian immunodeficiency virus (SIV) infection in intestinal mucosa is related to epithelial homeostasis disturbance and intestinal enteropathy [11].

Meanwhile, host cellular miRNAs can modulate HIV-1 infection by targeting either the conserved regions of HIV-1 genome or host gene transcripts, and these miRNAs may play pivotal roles in maintaining viral latency and promoting host defense [12], [13], [14]. HIV-1 *nef* appears to be the most widely focused gene for studying binding with miRNAs. The highly expressed cellular miRNAs miR-125b, miR-150, miR-28, miR-223 and miR-382 repress HIV-1 replication by targeting *nef* 3′-long-terminal repeat (LTR) region and contribute to viral latency in resting CD4^+^ T lymphocytes [15]. miR-29a specifically targets HIV-1 *nef* transcription and reduces viral production and infectivity, enhances HIV-1 mRNA association with RNA-induced silencing complexes, and sequesters viral mRNA in P bodies for further degradation [16], [17], [18], [19]. A cluster of other host miRNAs, such as miR-15a, miR-15b, miR-16, miR-224-3p, miR-223 and miR-24, have been studied *in silico* and predicted to bind with the HIV-1 *nef* 3′-LTR region [19]. Alternatively, some miRNAs regulate HIV-1 infection by targeting host gene transcripts. The differential regulation of cellular miR-148 on HLA-C alleles is associated with HIV control [20], [21]. Conversely, certain host cellular miRNAs appear to be essential for HIV to establish infection. Cellular miR-132 is upregulated in activated CD4^+^ T cells and potentiates HIV-1 replication by targeting host factor MeCP2 (Methyl-CpG binding protein 2) [22]. miR-217 and miR-34a are reported to favor Tat-induced HIV-1 LTR-driven transactivation by downregulating SIRT1 (sirtuin 1), a host-cell-encoded class II deacetylase [23], [24]. Recently, a novel HIV-1-encoded miRNA miR-H3 was identified by computational prediction and deep sequencing. miR-H3 is located in the mRNA region encoding the active center of reverse transcriptase and targets the HIV-1 5′-LTR for upregulating promoter activity and viral transcription [25]. Understanding these roles of miRNA in HIV-1 replication will be helpful to elucidate host-mediated antiviral response and explore new antiviral strategies.

Primary monocytes are refractory to HIV-1 infection and become permissive upon differentiation into macrophages or dendritic cells (DCs) [26], [27], [28], [29]. Multiple inefficiencies in several post-entry steps of the HIV-1 life cycle, such as reverse transcription, nuclear import of pre-integration complex, and viral translation, have been shown to be responsible for HIV-1 restriction in monocytes [29], [30], [31], [32]. The post-entry restriction of HIV-1 may be due to the existence of potential restriction factors or the absence of virus-dependent host factors. Low abundance of thymidine phosphorylase that is associated with a limited stock of dTTP contributes to refractory HIV-1 reverse transcriptase [28], and enrichment of host restriction factors, such as APOBEC3G/F (apolipoprotein B mRNA-editing, enzyme-catalytic, polypeptide-like 3G/F) [33] and SAMHD1 (SAM domain and HD domain-containing protein 1) [34], [35], [36], [37], are associated with HIV-1 restriction in monocytes or myeloid cells. miRNAs have also been reported to modulate HIV-1 infection in monocytes [38], [39], [40]. The abundance of miRNA-198 can repress the expression of cyclin T-1, and inhibit viral transcription in primary monocytes [41].

To uncover the restriction of HIV-1 replication by miRNAs in undifferentiated monocytes, we analyzed the miRNA expression profile in monocytes by miRNA-chip array and compared it with that of their differentiated monocyte-derived DC (MDDC) counterparts. We have recently reported that the translation of host protein pur-alpha was repressed by cellular miRNAs to inhibit HIV-1 infection in monocytes [39]. Here, we report that another host factor, namely VprBP, could also be targeted by cellular miRNA to modulate monocyte/MDDC susceptibility to HIV-1 infection.

## Results

### VprBP expression is essential for promoting HIV-1 infection

**Figure 1:**
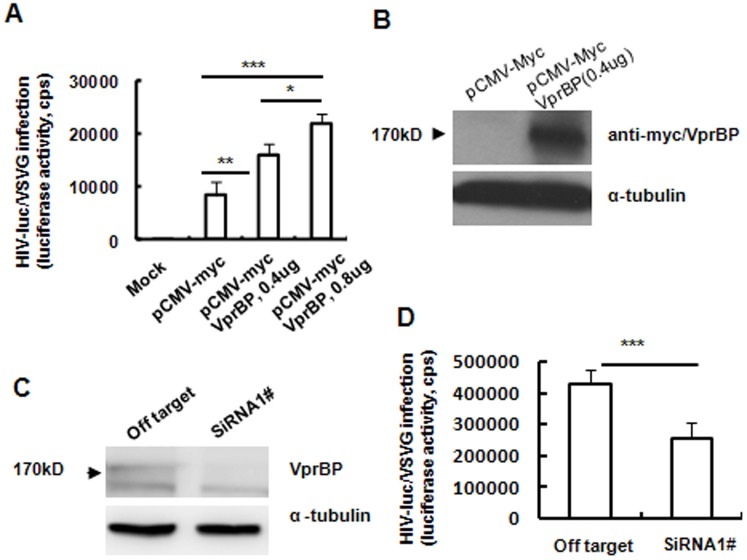
VprBP expression is essential for efficient HIV-1 infection. (A, B) Over-expression of VprBP facilitates viral infection. HEK293T cells were transfected with CMV-myc-VprBP plasmid or empty vector for 2 days and then infected by HIV-luc/VSV-G for an additional 2 days, and viral infection was measured. Exogenous expression of VprBP was confirmed by Western blotting. (C, D) Knockdown of VprBP inhibited HIV-1 infection. Magi/CCR5 cells were transfected with specific or off-target siRNA, and VprBP expression at the protein level and viral infection were measured. Data are mean ± SD. Results are representative of three independent experiments. *P<0.05, **P<0.01 and ***P<0.001, were considered significant differences in paired Student *t* test. cps, counts per second.

We investigated the importance of host factor VprBP in HIV-1 infection. We exogenously expressed VprBP by transfecting pCMV-myc-VprBP into HEK293T cells. As expected, overexpression of VprBP, as detected by Western blotting, significantly enhanced the infection of HIV-luc-Vpr^+^/vesicular stomatitis virus (VSV)-G in a dose-dependent manner (Figure 1A and B). Conversely, knocking down expression of VprBP in Magi/CCR5 cells with specific VprBP siRNA, as confirmed by Western blotting (Figure 1C), impaired the infection of HIV-luc-Vpr^+^/VSV-G (Figure 1D). VprBP appeared to promote HIV-1 PIC (Pre-integration Complex) post-nuclear import event, because the knocking-down of VprBP in Magi/CCR5 cells did not diminish the HIV-1 products of both late reverse transcripts (Late RT) and 2-LTR, and on the other hand, the overexpression of VprBP in 293T cells did not significantly enhance the products of both HIV-1 Late RT and 2-LTR (data not shown). These data demonstrate that VprBP expression is important for efficient HIV-1 infection.

### VprBP enhances HIV-1 infection in MDDCs in the presence of Vpr

**Figure 2:**
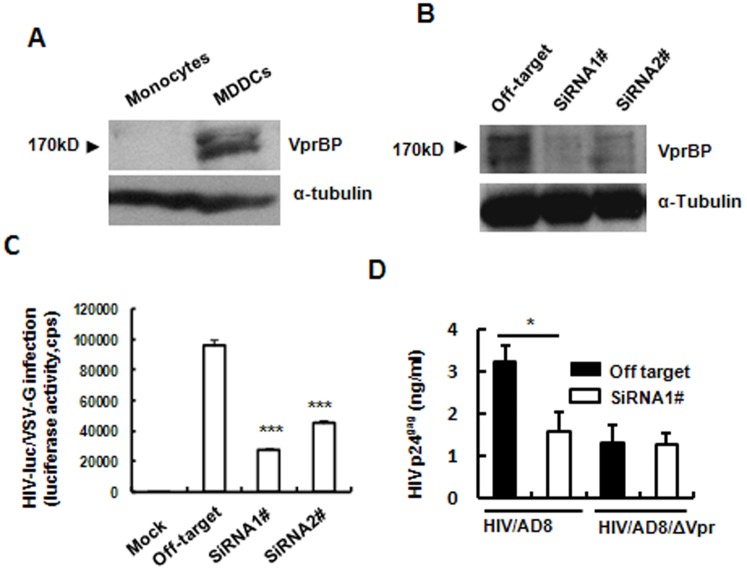
VprBP enhances HIV-1 infection in MDDCs. (A) VprBP expression in MDDCs and monocytes was detected by Western blotting. (B–D) Effect of knocking down VprBP on HIV-1 infection of MDDCs. MDDCs were transfected with specific siRNA 1^#^ and 2^#^ or off-target siRNA, and knockdown of VprBP was confirmed by Western blotting at 48 h post-transfection (B); transfected MDDCs were infected by HIV-luc/VSV-G for an additional 5 days, and viral infection was detected by measuring luciferase activity; alternatively, (C) transfected cells were infected for an additional 5 days by wild-type or *vpr*-deficient replication-competent HIV-1/AD8, and viral replication was monitored by quantifying p24^gag^ in culture supernatant. Data are mean ± SD. Results are representative of three independent experiments. *P<0.05 and ***P<0.001, were considered significant difference in paired Student *t* test.

To demonstrate further the importance of VprBP expression in promoting HIV-1 infection, we investigated VprBP expression in MDDCs. VprBP showed adequate expression in MDDCs compared with monocytes, as detected at the protein level (Figure 2A). When VprBP expression in MDDCs was interfered by specific siRNA (Figure 2B), the infection of pseudotyped single-cycle infectious HIV-luc-Vpr^+^/VSV-G was significantly impaired, demonstrating the crucial role of VprBP for efficient HIV-1 infection in primary MDDCs (Figure 2C).

VprBP is believed to be tightly conjugated with Vpr for functioning, for example, HIV-1 Vpr or HIV-2/SIVsmm/mac Vpx can interact with VprBP and assembles with DDB1 (DNA damage-binding protein 1) to form an E3 ubiquitin ligase complex, which targets cellular substrates for proteasome-mediated degradation and G2 cell-cycle arrest [42], [43], [44]. To investigate the dependence of Vpr for VprBP-promoting HIV-1 infection, we used Vpr-deficient HIV-1. Enhanced HIV-1 replication was only observed in Vpr-containing viruses, and knocking-down of VprBP did not impair the infection of *vpr*-deficient replication-competent HIV-1/AD8 in MDDCs (Figure 2D). The results demonstrate that promotion of VprBP in HIV-1 infection is Vpr dependent, and these results also confirm the VprBP dependence of Vpr-promoted HIV-1 infection. These data demonstrate the importance of VprBP in HIV-1 infection in MDDCs and the dependence on Vpr of VprBP-promoted viral infection.

### Cellular miR-1236 targets 3′-untranslated region (UTR) of VprBP mRNA for translation inhibition in monocytes

**Figure 3:**
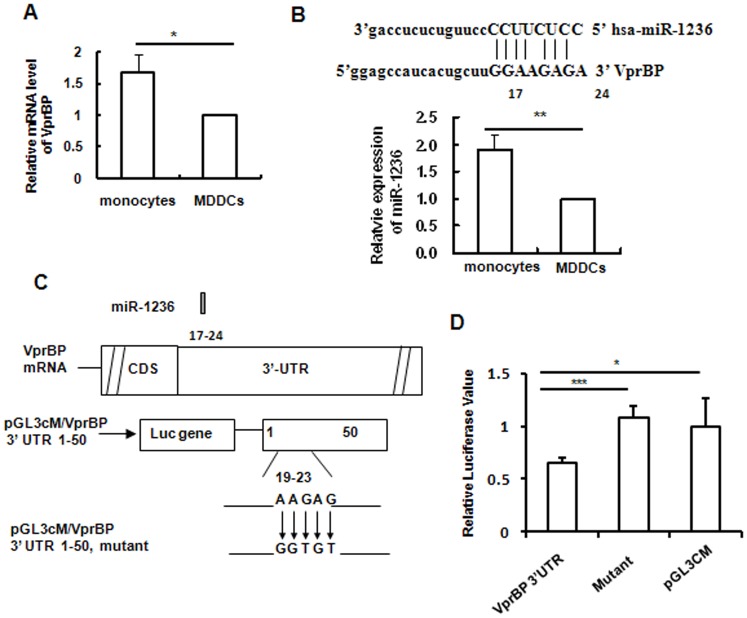
3′-UTR of VprBP mRNA is targeted by miR-1236. (A) Relative level of VprBP mRNA in monocytes compared with MDDCs. Total cellular RNAs were extracted from cells and the VprBP mRNA was measured with SYBR green-based semi-quantified real-time (RT-) PCR and normalized with β-actin. (B) Sequences of miR-1236 and alignments with the conserved binding position of 3′-UTR of VprBP, and the relative expression of miR-1236 in monocytes compared with MDDCs was quantified with Bulge-loop miRNA RT-PCR. (C) Schematic diagram showing binding of miR-1236 on 3′-UTR of VprBP mRNA and the point mutation of conserved positions of target sequences. (D) Inhibition of miRNA mimics on the expression of pGL3cM containing the 3′-UTR fragment of VprBP mRNA or mutant within conserved binding position. Data are mean ± SD. Results are representative of three independent experiments. *P<0.05, **P<0.01 and ***P<0.001, were considered significant difference in paired Student *t* test.

We demonstrated above that VprBP was not expressed in monocytes, as detected at the protein level (Figure 2A). To elucidate the underlying mechanism for repression of VprBP expression in primary monocytes, VprBP transcription was further assessed. Unexpectedly, monocytes expressed even higher levels of VprBP mRNA transcripts than did MDDCs (Figure 3A). This indicates post-transcriptional inhibition of VprBP expression in monocytes.

Considering the regulatory role of miRNA on translation, we compared the miRNA expression in monocytes and MDDCs by Agilent miRNA-chip array. Target prediction of miRNAs and alignment of miRNAs with target sequences were achieved using TargetScan. We found the 17–24 bases of VprBP 3′-UTR were targeted by hsa-miR-1236. Expression of miR-1236 was verified with Bulge-loop miRNA qRT-PCR Primer Sets. The expression was adjusted based on U6 small nuclear RNA transcription. miR-1236 expression was significantly enhanced in monocytes compared with MDDCs (Figure 3B).

To confirm the targeting of VprBP mRNA 3′-UTR by miRNA-1236, 3′-UTR fragments of VprBP mRNA that contained the conserved binding positions were cloned into pGL3cM, and mutations were introduced at the conserved binding positions of VprBP 3′-UTR to reduce Watson–Crick base pairing with miRNAs (Figure 3C). Inhibition of transfected miRNA mimics on expression of pGL3cM-containing 3′-UTR fragments of VprBP or mutants was quantified using a dual-luciferase reporter assay system, and the relative activity of firefly and *Renilla* luciferases was calculated. Transfection with miRNA mimics significantly inhibited expression of pGL3cM/VprBP 3′-UTR, and the mutation of conserved binding positions abolished the inhibition mediated by miRNA mimics (Figure 3D). These data demonstrated that miR-1236 targets VprBP mRNA 3′-UTR for repression in monocytes.

### VprBP-targeted miR-1236 modulates differentiation-dependent susceptibility of monocytes/MDDCs to HIV-1 infection

**Figure 4:**
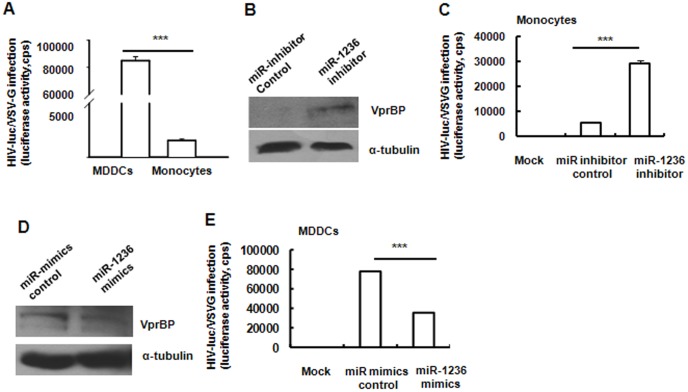
miR-1236 modulates HIV-1 infection by targeting VprBP. (A) Comparison of the infection of HIV-luc/VSV-G in MDDCs versus in monocytes. (B, C) Transfection of monocytes with miR-1236 inhibitor promoted VprBP translation and significantly enhanced infection by HIV-luc/VSV-G. (D, E) Transfection of MDDCs with miR-1236 mimics impaired translation of VprBP and significantly inhibited infection by HIV-luc/VSV-G. Results are representative of three independent experiments. Data are mean ± SD. ***P<0.001 was considered significant difference in paired Student *t* test.

HIV-1 infection was restricted in primary monocytes, but this restriction could be partially relieved when monocytes were differentiated into DCs upon stimulation with cytokines granulocyte–macrophage colony-stimulating factor and interleukin-4, as confirmed in Figure 4A. Multiple causes have been provided to reveal the differentiation-dependent susceptibility of monocytes to HIV-1 infection [29], [30], [31], [32], [39].

Given the important role of VprBP in promoting HIV-1 infection and the regulation of VprBP translation by miR-1236, we were interested in whether miR-1236 modulated monocyte/MDDC susceptibility to HIV-1 infection. We transfected monocytes with chemically synthesized, single-stranded miRNA inhibitors. As shown in Figure 4B and C, the transfection of miR-1236 inhibitors enhanced the translation of VprBP in monocytes and significantly promoted the infection of HIV-Luc/VSV-G. Complementarily, when the chemically synthesized miR-1236 mimics were transfected into MDDCs, translation of VprBP was suppressed (Figure 4D), and infection of MDDCs by HIV-Luc/VSV-G was accordingly significantly diminished (Figure 4E). These data demonstrate that miR-1236-mediated regulation of VprBP expression modulates the differentiation-dependent susceptibility of monocytes/MDDCs to HIV-1 infection.

## Discussion

HIV-1 depends on host-cell-encoded factors for completing its life cycle, and hundreds of HIV-1-dependent host genes for replication have been uncovered by uniformly conducted in HIV-1 non-natural biology target cells or cell lines [45], [46], [47]. By adopting more physiologically relevant primary cells, we screened gene expression in monocytes by mRNA and miRNA microarray analysis, and compared it with that in their differentiated MDDCs [39]. Our purpose was to define on a large scale the differently expressed genes that could modulate HIV-1 susceptibility in monocytes/MDDCs.

VprBP, also known as DCAF1 (DDB1–CUL4-associated factor 1), is a component that forms a CUL4–DDB1–VprBP/DCAF1 E3 ubiquitin protein ligase complex. As the Vpr binding protein, VprBP is tightly conjugated with Vpr for functioning [48], [49]. Here, we demonstrated that VprBP and Vpr showed mutual dependence in promoting HIV-1 infection, although the detailed mechanisms need to be clarified.

VprBP functions as the substrate recognition module within CUL4–DDB1–VprBP/DCAF1 E3 ubiquitin protein ligase complex and bridges target proteins to DDB1. HIV-1 Vpr or HIV-2/SIVsmm/mac Vpx can interact with VprBP and assembles with DDB1 to form an E3 ubiquitin ligase complex, which targets cellular substrates for proteasome-mediated degradation and G2 cell-cycle arrest [42], [43], [44], [50]. A recent study has reported that the direct interaction of HIV-1 Vpr with structure-specific endonuclease regulator SLX4 recruits VprBP and kinase-active PLK1, and enhances cleavage of DNA by SLX4-associated MUS81–EME1 endonucleases, resulting in G2/M arrest [51]. Moreover, the DDB1–Cul4–DCAF1/VprBP E3 ubiquitin ligase complex appears essential for HIV1 Vpr-mediated degradation of the uracil-DNA glycosylases 2 and SMUG1 (single-strand-selective monofunctional uracil-DNA glycosylase 1) [52]. It has been reported that DCAF1/VprBP is recruited by HIV-2/SIVsmm/mac Vpx to hijack the CUL4–DDB1 E3 ubiquitin ligase complex for degradation of host-restrictive factor SAMHD1 [34], [35], [53], facilitating HIV-1 infection in myeloid cells. However, SAMHD1 shows comparable expression in monocytes compared with MDDCs, suggesting that VprBP-facilitated HIV-1 infection in MDDCs is not attributed to the SAMHD1 degradation induced by CUL4–DDB1–DCAF1/VprBP E3 ubiquitin protein ligase complex.

In our miRNA-chip analysis, we also found that other cellular miRNAs, such as miR-15a, miR-15b, miR-16, miR-93, miR-106b and miR-20a, can inhibit HIV-1 infection in monocytes, and Pur-alpha, a host-cell-encoded transcription factor required for enhancing Tat-derived viral transactivation, was targeted for repression in monocytes [39].

In summary, our results demonstrate that host factor VprBP was targeted by cellular miR-1236 to modulate monocyte/MDDC differentiation-dependent susceptibility to HIV-1 infection. miRNA-based therapies are being developed, and understanding the modulation of HIV-1 infection by cellular miRNAs may provide key small RNAs or the identification of new important protein targets regulated by miRNAs for antiviral therapeutic interventions.

## Materials and Methods

### Cell

Human peripheral blood mononuclear cells (PBMCs) from healthy donor were purchased from Blood Center of Shanghai (Shanghai, China). CD14^+^ monocytes were isolated from PBMCs by using CD14 antibody-coated magnetic beads (Miltenyi Biotec, Germany) as previously described [54]. Monocytes were differentiated into MDDCs by stimulation with 50 ng/ml of GM-CFS and IL-4 for 6 days. HEK293T cell line was kindly gifted by Dr. Li Wu (The Ohio State University, Columbus, OH, USA) and HeLa-cell-derived Magi/CCR5 cell line[39] was a gift from Dr. Paul Zhou (Institute Pasteur of Shanghai, Chinese Academy of Sciences, Shanghai, China).

### Virus stock and infection assay

Single-cycle HIV-Luc/VSV-G was produced by calcium-phosphate-mediated co-transfection of HEK293T cells with the luciferase reporter HIV-1 proviral vector NL-Luc-E^-^R^+^(Vpr^+^) and an expression plasmid of vesicular stomatitis virus G (VSV-G) protein. Replication competent HIV-1-AD8 (R5 tropic) virus or Vpr deletion AD8 virus were obtained by transfecting HIV-1 vectors of pNLAD8 or pNLAD8-ΔVpr, respectively. Plasmids were kindly provided by Dr. Li Wu (The Ohio State University, Columbus, OH, USA). Harvested supernatants that contained viral particles were filtered and quantified with p24^gag^ capture ELISA. Cells were infected with HIV-1-Luc/VSVG (1 ng p24^gag^) or replication competent HIV-1 (1 ng p24^gag^) for 2 h, and after washing, cells were further cultured for 2 days (cell line) or 5 days (MDDCs and monocytes). Viral infection was detected by measuring the luciferase activity from the cell lysates or detecting the p24^gag^ level from the supernatant. The HIV-1 p24^gag^ specific monoclonal antibodies are kindly gift of Prof. Yong-Tang Zheng, from Kunming Institute of Zoology, Chinese Academy of Sciences.

### Transfection of plasmids, siRNA, miRNA inhibitor or miRNA mimics

Cells were transfected by Lipofectamine 2000 (Invitrogen) with plasmids of pCMV-myc/pCMV-myc-VprBP, or with specific duplex siRNA of VprBP or off-target control (GenePharma, Shanghai, China), or with miR-1236 inhibitor or mimics (GenePharma). Then cells were either collected to detect the condition of expression by Western blotting, or for viral infection as described above. The sequences of siRNA duplex, miRNA inhibitors and mimics were as follows: VprBP siRNA 1# 5′-GGCCCAGAUAACCGAAUAUTT-3′, VprBP siRNA 2# 5′-GCGACUCAUUC UCCAAUAUTT-3′. Plasmid pCMV-myc-VprBP encoding full-length myc-tagged VprBP (also known as DCAF1) was described previously[50]. For Western blotting, the anti-myc monoclonal antibody (clone 19C2, Abmart, Shanghai, China) and rabbit polyclonal antibody against VprBP (Santa cruz, America) were used.

### MiRNA and miRNA microarray

The mRNA and miRNA expression profiles during monocyte to DC differentiation were screened with Affymetrix U133 plus 2.0 array and Agilent miRNA expression array as described previously [39], respectively. Genes were analyzed further online with the functional annotation tool in DAVID Bioinformatics Resources 6.7 (National Institute of Allergy and Infectious Diseases, U.S. National Institute of Health, Bethesda, MD, USA). Target prediction of miRNAs and the alignments of miRNAs with target sequences were done by using TargetScan (http://www.targetscan.org). The expression of miRNAs was verified with Bulge-loop miRNA qRT-PCR Primer Sets (RiboBio Corp, Guangzhou, China). The expression was adjusted based on U6 small nuclear RNA transcription, and relative miRNA expression of indicated controls was calculated. PCR was performed on the ABI 7900HT Real-Time PCR system (Applied Biosystems, Foster City, CA, USA).

### Construct and luciferase report assay

VprBP 3′ UTR was amplified from the total RNA extracted from the HEK293 cell line. Binding sites of VprBP 3′ UTR for miR-1236 were synthesized as follows: 5′-CGGAGCCATCACTGCTTGGAAGAGATTCTTGGCAGAGAGAAGAGGGGACAA-3′ (forward), 5′-GATCTTGTCCCCTC TTCTCTCTGCCAAGAATCTCTTCCAAGCAGTGATGGCTCCGAGCT-3′ (reverse). And point mutations of conserved binding sites of VprBP 3′-UTR for miR-1236 were synthesized as follow: 5′-CGGAGCCATCACTGCTTAACGA CAGTTCTTGGCAGAGAGAAGAGGGGACAA-3′ (forward), 5′-GATCTTGTCC CCTCTTCTCTCTGCCAAGAACTGTCGTTAAGCGTGATGGCTCCGAGCT-3′ (reverse). MiRNA binding reporter pGL3CM-VprBP-3′UTR or mutation was constructed with Bgl II and Sac I. Plasmid pGL3CM was kindly gifted by Dr. Ke Lan (Institute Pasteur of Shanghai, Chinese Academy of Sciences, Shanghai, China).

A Dual-Luciferase Reporter Assay System (Promega, Madison, WI, USA) was used to examine the effects of miRNAs on their target genes as described previously[39]. Briefly, pGL3CM reporter plasmids (100 ng) were co-transfected into HEK293T cells with miR-1236 (1μl) or off target control and 10 ng pRL-TK. Lipofectamine 2000 was used for transfection. Cells were collected for luciferase assay 48 h post transfection and were lysed with 100 ul 1× negative lysis buffer (Promega) for 15 min; Lysate (20 µl) was added with Luciferase Assay Buffer and mixed to measure firefly luciferase on a Veritas luminometer (Turner BioSystems, Sunnyvale, CA, USA). Then Stop & Glo Reagent (30 µl) was added and mixed to detect renilla luciferase.

### Statistical analysis

Statistical analysis was performed using a paired t test with SigmaStat 2.0 (Systat Software, San Jose, CA, USA).
